# ﻿Synonymization of two, monotypic black-coral-commensal scale worm genera, *Antipathipolyeunoa* Pettibone, 1991 and *Parahololepidella* Pettibone, 1969 (Polynoidae, Aphroditiformia)

**DOI:** 10.3897/zookeys.1178.106101

**Published:** 2023-09-01

**Authors:** Brett C. Gonzalez, Victor M. Conde-Vela, Karen J. Osborn

**Affiliations:** 1 Smithsonian Institution, National Museum of Natural History, Department of Invertebrate Zoology, P.O. Box 37012, Washington D.C., USA National Museum of Natural History, Department of Invertebrate Zoology Washington United States of America

**Keywords:** Annelida, Antipatharia, black corals, polynoid, *Tanacetipathes*

## Abstract

*Parahololepidella* Pettibone, 1969 is a polynoid genus commensal with the antipatharian genus *Tanacetipathes* Opresko, 2001. These scale worms are elongate with numerous segments and small elytra. To date, the only other known polynoid associated with *Tanacetipathes* is *Antipathipolyeunoa* Pettibone, 1991. By re-examining the holotype of *Antipathipolyeunoa*, we have identified several overlooked characters that no longer distinguish this genus from *Parahololepidella*. Based on the presence of chaetae on the tentacular segment and elytral irregularity on posterior segments, we propose synonymizing *Antipathipolyeunoa* with *Parahololepidella*.

## ﻿Introduction

Polynoidae Kinberg, 1856 scale worms are one of the most diverse groups of Annelida found in association with other organisms (Martin and Britayev 2018). Members of this hyperdiverse group are commonly found in association with cnidarians (e.g., Barnich et al. 2013), echinoderms (e.g., Sugiyama et al. 2020), decapods (e.g., Pettibone 1993), and even other annelid hosts (e.g., Britayev and Martin 2006). Notably, nearly 50% of known commensal polynoids are associated with black corals and octocorals (Martin and Britayev 1998; Buhl-Mortensen and Mortensen 2004). However, the limited knowledge of the taxonomy and biology of these scale worms restricts our understanding of their adaptations to their hosts (Molodtsova et al. 2016; Martin and Britayev 2018).

Most black-coral-commensal scale worms have elongate bodies with more than average numbers of segments and elytra. Despite these gross similarities, the highly specialized nature of commensal polynoids associated with antipatharian corals is reflected in the erection of separate polynoid genera for each different host (e.g., Pettibone 1969, 1991). In 1969, Pettibone revisited *Polyeunoa* McIntosh, 1885 and *Hololepidella* Willey, 1905—two similar and often confused genera of elongate polynoids (Pettibone 1969). As a result, *Polyeunoa* and *Hololepidella* were revised and three new genera were erected, including *Parahololepidella* Pettibone, 1969, which has been characterized by numerous segments (≥140) and elytra, elytra irregularly arranged posteriorly with different numbers on right and left sides, tentacular segment (segment 1) with chaetae, neuropodia with a digitiform subacicular process, and slightly hooked neurochaetae thicker than notochaetae (Pettibone 1969; Fauchald 1977; Britayev et al. 2014). The only known species, *Parahololepidellagreeffi* (Augener, 1918) from West Africa, was originally reported as a free-living species inhabiting mucous tubes incrusted with sand grains and shell fragments. Pettibone (1969) concluded that these tubes were likely associated with another commensal organism and not the worm in question. Britayev et al. (2014) redescribed *Parahololepidellagreeffi* from the syntypes and newly collected topotypes from São Tomé and Príncipe (Gulf of Guinea), finding specimens living in association with the antipatharian Tanacetipathescf.spinescens and not free-living as originally thought (Rullier 1964; Pettibone 1969). These newly collected worms were camouflaged along the main stems of the coral branches, agreeing with Pettibone’s (1969) assessment that this species did not live in mucous tubes.

In 1991, Pettibone erected three monotypic genera commensal with antipatharian corals, *Antipathipolyeunoa*, *Bayerpolynoe* and *Tottonpolynoe*, and included a key to all related taxa (Pettibone 1991). Among these new genera, *Antipathipolyeunoa* Pettibone, 1991 was described from specimens removed from the stems of *Tanacetipathestanacetum* (Pourtalès, 1880) [as *Antipathes*] in Barbados (Pettibone 1991). The only known species, *Antipathipolyeunoanuttingi* Pettibone, 1991, was characterized by numerous segments and elytra, tentacular segment (segment 1) without chaetae, neuropodia with projecting subacicular process, and falcate neurochaetae stouter than notochaetae. Pettibone (1991) remarked that *Antipathipolyeunoa* closely resembled *Polyeunoa* (*sensu*Pettibone 1969), but differed based on the presence of a prominent subacicular process on the prechaetal acicular lobe of the neuropodium. Surprisingly, the included key and remarks both overlooked her previous work on *Parahololepidella* despite its striking resemblance to *Antipathipolyeunoa*, specifically, in the presence of a prominent subacicular process (Pettibone 1969, 1991). This omission could be attributed to the fact that, at that time, *Parahololepidellagreeffi* was not known to be associated with black corals (Britayev et al. 2014).

When recently comparing the original diagnoses of *Parahololepidella* and *Antipathipolyeunoa*, it became evident that the only distinguishing feature between these two genera was the presence/absence of chaetae on segment 1. Pettibone (1969) indicated that the posterior elytral pattern may have “some irregularity” in *Parahololepidella*, but did not describe this condition further, and made no reference to this condition for *Antipathipolyeunoa*. Pettibone (1969) noted that the exact elytral arrangement was often omitted in elongate polynoids given their variability, but appeared constant for the anterior regions. The later redescription of *Parahololepidellagreeffi* (see Britayev et al. 2014) described in detail the irregular and asymmetrical arrangement of elytra, sometimes occurs from segment 32, where a single dorsal cirrus and an elytron may occur on opposing sides of the same segment.

Upon re-examination of the holotype (USNM 80097) and the three paratypes (USNM 136587) of *Antipathipolyeunoanuttingi*, several morphological features were observed that were missed during the original description. We found that all specimens of *A.nuttingi* have chaetae on the tentacular segment (segment 1), and elytral irregularities are present in the posterior region of all three paratypes. The lack of elytral variation in the holotype suggests that this condition may have been overlooked because it is the only specimen complete with dorsal cirri and elytra still attached.

Based on the presence of chaetae on the tentacular segment and the irregularities in the posterior segments found in the type material, we conclude that no significant differences exist between the monotypic genera *Parahololepidella* and *Antipathipolyeunoa*. Therefore, we propose the synonymy of *Antipathipolyeunoa* with *Parahololepidella* and accordingly include *Antipathipolyeunoanuttingi* as a member of *Parahololepidella*; providing an updated systematic account for *Parahololepidella* as well as additional morphological details for *Parahololepidellanuttingi* comb. nov.

## ﻿Systematics

**Suborder Aphroditiformia Levinsen, 1883**

**Family Polynoidae Kinberg, 1856**

### ﻿Subfamily Arctonoinae Hanley, 1989

#### ﻿ Parahololepidella

Taxon classificationAnimaliaPhyllodocidaPolynoidae

Pettibone, 1969

96221E9E-4D7D-58CA-9A80-7CCD543BC2B5

Parahololepidella Pettibone, 1969: 54 [type species: Hololepidellagreeffi Augener, 1918, by original designation].—Britayev et al. 2014: 28 [diagnosis emended; type species redescribed using syntypes (ZHM 5692) and topotypes (NNMN 24481) due to the poor state of the original syntypes].
Antipathipolyeunoa Pettibone, 1991: 715 [type species: Antipathipolyeunoanuttingi Pettibone, 1991, by original designation].

##### Remarks.

The type specimens of *Parahololepidella* (ZHM 5692) were examined and illustrated by Pettibone for her 1969 revision of *Hololepidella*, and subsequently re-examined by Britayev et al. (2014) when they redescribed *Parahololepidellagreeffi* (NNMN 24481 and MNCN 16.01/13708). Only original notes and illustrations by Pettibone were examined herein.

*Parahololepidella* now includes two species commensal with the antipatharian genus *Tanacetipathes* Opresko, 2001. Both *Parahololepidella* species show cryptic coloration patterns and are found nestled along the stems of the coral branches.

#### ﻿ Parahololepidella nuttingi

Taxon classificationAnimaliaPhyllodocidaPolynoidae

(Pettibone, 1991) comb. nov.

E274653B-2D98-5C90-AA2F-70614B0FC839

Fig. 1

Antipathipolyeunoa nuttingi Pettibone, 1991: 716–719, figs 1, 2.

##### Material examined.

***Holotype*. Barbados** ● 1; Sta. 65, off Payne’s Bay Church; 91 m; 1918; collector CC Nutting; Barbados-Antigua Expedition; on *Antipathestanacetum* (now *Tanacetipathes*); USNM 80097. ***Paratypes*. Venezuela** ● 3; Sta. 736, W of Tortuga Island; 10.95, −65.8667; 69–155 m; 22 July 1968; *R/V* Pillsbury; on *Antipathestanacetum* (now *Tanacetipathes*); USNM 136587.

##### Redescription

**Figure 1.**
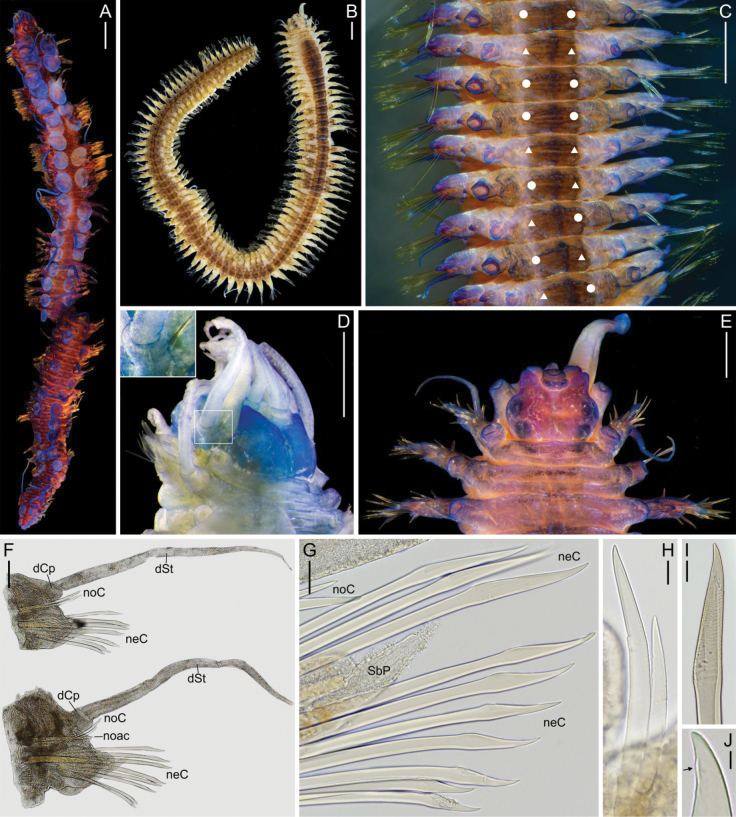
*Parahololepidellanuttingi* (Pettibone, 1991) comb. nov., holotype of *Antipathipolyeunoanuttingi* USNM 80097 (**A, D, F–J**); paratype USNM 136587 (**B, C, E**) **A, B** whole specimens, dorsal view **C** dorsal view of middle segments showing variation in elytral distribution; white circles represent dorsal cirrophores and white triangles represent elytrophores **D** dorsolateral view of anterior with detail (inset) of left tentacular segment (segment 1) showing single chaeta **E** anterior end, dorsal view **A, C–E** specimens stained with Shirlastain A to observe morphology in detail **F** cirrigerous parapodia, ventral view, ventral cirri missing **G** chaetae from cirrigerous parapodium **H** notochaetae **I** distal end of supraacicular neurochaetae **J** tip of supraacicular neurochaeta with tooth-like protuberance indicated with black arrow. Abbreviations: dCp, dorsal cirrophores; dSt, dorsal style; neC, neurochaetae; noac, notoacicula; noC, notochaetae; SbP, subacicular process. Scale bars: 1 mm (**A–C**); 0.5 mm (**D, E**); 0.2 mm (**F**); 50 μm (**G**); 20 μm (**H, I**); 5 μm (**J**).

**(based on the holotype).** Body with numerous segments, >80 (Fig. 1A, B). Elytra numerous, >40 pairs. Paired elytra on segments 2, 4, 5, 7, 9, 11, 13, 15, 17, 19, 21, 23, 26, 29, 32, 33, 35, then alternating dorsal cirri and elytra until the end (holotype; Fig. 1A). Elytral variation present in posterior third of paratypes (see “Variation” below); elytron and dorsal cirrus may occur on same segment (Fig. 1C). Elytra oval; margins and surface smooth; cover dorsum anteriorly; dorsum exposed on middle and posterior segments (Fig. 1A). Elytrophores short; less pronounced than dorsal cirrophores. Dorsal tubercles inconspicuous. Dorsal cirrophores not extending beyond notopodia (Fig. 1F). Dorsal cirrostyles smooth, 5–6 times longer than parapodium.

Prostomium bilobed; anterior notch shallow (Fig. 1D, E). Cephalic peaks prominent, subtriangular, as continuous extension of the prostomium, equal width to median antenna ceratophore (Fig. 1D, E). Median antenna in anterior notch (Fig. 1D, E); ceratophore short, bulbous with smooth ceratostyle, roughly three times the width of the prostomium. Lateral antennae inserted subterminally; ceratophores distinct (Fig. 1D); styles shorter than palps. Palps short, stout, ~1.5 times the width of the prostomium (Fig. 1B, D, E). Two pairs of eyes; large, laterally positioned. Tentacular segment (segment 1) not visible dorsally (Fig. 1E). Dorsal tentacular cirri longer than ventral tentacular cirri. Tentaculophores lateral to prostomium, with single chaeta (Fig. 1D, inset); tentacular styles smooth. Facial tubercle oblong, with smooth rounded margins. Segment 2 (buccal segment) with first pair of elytra and biramous parapodia; nuchal fold absent. Buccal cirri longer than following ventral cirri. Dorsal cirri from segment 3.

Parapodia subbiramous. Notopodia reduced, with subconical lobe (Fig. 1F). Neuropodia broad; subconical prechaetal lobe longer than postchaetal lobe, with digitiform subacicular process (Fig. 1F, G). Noto- and neuroacicula penetrating epidermis on some segments (Fig. 1F). Notochaetae few (9–2) (Fig. 1F), decreasing posteriorly, less stout than neurochaetae (Fig. 1G, H). Neurochaetae few (12–6) (Fig. 1F, G), more numerous in middle segments; shafts smooth (Fig. 1G); faint spinous rows distally (Fig. 1I); tips falcate, 4–7 times longer than wide (Fig. 1G, I), occasionally with small, tooth-like protuberance (Fig. 1J). Ventral cirri from segment 3, smooth, longer than neuropodium in anterior segments, becoming subequal in length posteriorly. Pygidium rounded. Anus terminal. Anal cirri long, equal in length of last five segments. Nephridial papillae present from segment 6.

##### Measurements.

Fixed holotype 21.5 mm long, 2.2 mm wide excluding chaetae, 83 segments. The three paratypes (USNM 136587) consist of one posteriorly incomplete specimen, two additional anterior ends, and several middle and posterior fragments. It was only possible to trace one of the two shorter anterior fragments to their respective remaining body fragments. Longest anterior paratype fragment, 24.5 mm long, 2 mm wide excluding chaetae, 85 segments. Reconstructed paratype, 25 mm long, 2.3 mm wide excluding chaetae, 102 segments. Shortest anterior paratype fragment, 5.7 mm long, 1.5 mm wide excluding chaetae, 25 segments.

##### Variation.

Pigmentation is present in all specimens, present along the midline of the dorsum, with wider bands of pigment present in the paratypes (Fig. 1B). Additional pigmentation occurs on the cirrophores (Fig. 1B), but it is otherwise completely lacking (Fig. 1B, C). Of the two nearly complete paratypes, the elytral distribution patterns are as follows:

1. Paratype 1.

- R: 2, 4, 5, 7, 9, 11, 13, 15, 17, 19, 21, 23, 26, 29, 32, 33, 35, 37, 38, 41, 43, 45, 47, 49, 52, 54, 56, 59, 61, 63, 65, 67, 69, 71, 73, 75, 77, 79, 81, 83, 85
- L: 2, 4, 5, 7, 9, 11, 13, 15, 17, 19, 21, 23, 26, 29, 32, 34, 36, 38, 41, 43, 45, 47, 49, 51, 53, 55, 56, 58, 59, 60, 62, 63, 65, 67, 69, 71, 73, 75, 77, 79, 81, 83, 85

1. Paratype 2.

- R: 2, 4, 5, 7, 9, 11, 13, 15, 17, 19, 21, 23, 26, 29, 32, 34, 36, 38, 40, 42, 44, 46, 47, 50, 52, 54, 56, 58, 60, 62, 64, 66, 86, 70, 72, 74, 76, 80, 82, 84, 86, 88, 90, 92, 94, 96, 98, 100, 102.
- L: 2, 4, 5, 7, 9, 11, 13, 15, 17, 19, 21, 23, 26, 29, 32, 33, 35, 37, 39, 41, 43, 45, 47, 49, 50, 52, 54, 56, 58, 60, 62, 64, 66, 68, 70, 72, 74, 76, 80, 82, 84, 86, 88, 90, 92, 94, 96, 98, 100, 102.

##### Remarks.

The current diagnosis mostly agrees with that of Pettibone (1991) but differs on the presence of chaetae on the tentacular segment (for both holotype and paratypes), and for the irregular distribution pattern of elytra found posteriorly in the paratypes. The holotype only varies from the paratypes in the elytral variation and pigmentation.

*Parahololepidellanuttingi* comb. nov. is very similar to *P.greeffi*, but can be differentiated as follows. In *P.nuttingi*, the dorsal cirrophores are shorter than the notopodium in middle segments, whereas in *P.greeffi*, the dorsal cirrophores surpass the notopodium (see Pettibone (1991, fig. 1G) and (1969, fig. 4C), respectively). In *P.nuttingi*, the neurochaetae have falcate tips 4–7 times longer than wide, whereas in *P.greeffi*, the falcate tips are only 2–3 times longer than wide. And finally, in *P.nuttingi*, when present, the tooth-like protuberance on the falcate tips of the neurochaetae are small (easily overlooked), whereas in *P.greeffi*, the tooth-like structures are larger when present, giving them almost a bidentate appearance. Britayev et al. (2014) cautioned that the neurochaetal tooth-like structures in *P.greeffi* (ZMH 5692) were artifacts due to poor preservation and dehydration over time. However, given that similar structures were found in all specimens of *P.nuttingi*, we feel that this character is valid and illustrates the importance of detailed microscopical examination and reexamination.

## Supplementary Material

XML Treatment for Parahololepidella

XML Treatment for Parahololepidella nuttingi

## ﻿Additional information

### Author ORCIDs

Brett C. Gonzalez https://orcid.org/0000-0001-6968-2677

Victor M. Conde-Vela https://orcid.org/0000-0002-3964-5426

Karen J. Osborn https://orcid.org/0000-0002-4226-9257

### Data availability

All of the data that support the findings of this study are available in the main text.
